# Genomic Sequencing of *Bordetella pertussis* for Epidemiology and Global Surveillance of Whooping Cough

**DOI:** 10.3201/eid2406.171464

**Published:** 2018-06

**Authors:** Valérie Bouchez, Julien Guglielmini, Mélody Dazas, Annie Landier, Julie Toubiana, Sophie Guillot, Alexis Criscuolo, Sylvain Brisse

**Affiliations:** Institut Pasteur, Paris, France

**Keywords:** Bordetella pertussis, whole-genome sequencing, multilocus sequence typing, nomenclature database, phylogeny, epidemiology, bacteria, pertussis, whooping cough, surveillance

## Abstract

*Bordetella pertussis* causes whooping cough, a highly contagious respiratory disease that is reemerging in many world regions. The spread of antigen-deficient strains may threaten acellular vaccine efficacy. Dynamics of strain transmission are poorly defined because of shortcomings in current strain genotyping methods. Our objective was to develop a whole-genome genotyping strategy with sufficient resolution for local epidemiologic questions and sufficient reproducibility to enable international comparisons of clinical isolates. We defined a core genome multilocus sequence typing scheme comprising 2,038 loci and demonstrated its congruence with whole-genome single-nucleotide polymorphism variation. Most cases of intrafamilial groups of isolates or of multiple isolates recovered from the same patient were distinguished from temporally and geographically cocirculating isolates. However, epidemiologically unrelated isolates were sometimes nearly undistinguishable. We set up a publicly accessible core genome multilocus sequence typing database to enable global comparisons of *B. pertussis* isolates, opening the way for internationally coordinated surveillance.

Whooping cough (or pertussis) is a vaccine-preventable disease caused mainly by the bacterium *Bordetella pertussis*, and to a lesser extent by *B. parapertussis*. The infection is most severe in infants who are too young to be vaccinated or are not yet fully vaccinated. The burden of disease is still high: 24 million pertussis cases and 160,700 deaths from pertussis in children <5 years of age in 2014 (*1*). The introduction of vaccination using whole-cell vaccines in the 1950s, and the switch to acellular vaccines targeting only some antigens in the 1980–1990s, have played a central role in the control of whooping cough. However, increasing incidence of the disease and large outbreaks have been reported recently in many countries (*2*–*5*). The observed resurgence of whooping cough underlines the need for reinforced surveillance of strain evolution, local spread, and global transmission. For example, the relative contributions of intercountry spread compared with local, independent evolution of strains that do not express pertactin (*6*–*8*), one of the components of acellular vaccines, are unknown. This gap limits our ability to interpret the local prevalence of pertactin-negative isolates and to define the effects of country-specific vaccine strategies on the emergence of antigen-deficient isolates. 

Until now, strain genotyping for surveillance and epidemiology has been based mostly on pulsed-field gel electrophoresis (PFGE), antigen and virulence factor genotyping, 7-gene multilocus sequence typing (MLST), or multilocus variable-number tandem-repeat analysis (MLVA) (*9*–*11*). PFGE achieves some level of resolution given the high structural dynamics of *B. pertussis* genomes, driven by insertion sequence elements dynamics (*12*), and is more discriminatory than MLVA or MLST. However, *B. pertussis* clinical isolates exhibit strong genetic homogeneity (*11*,*13*). Therefore, these traditional typing methods have largely failed to define local chains of transmission.

Whole-genome sequencing (WGS) provides the highest possible resolution of genetic differences among individual isolates. Working with WGS of an international collection of *B. pertussis* isolates collected through 2010, Bart et al. (*13*) provided a global phylogenetic structure of *B. pertussis* and analyzed genome evolutionary dynamics across the prevaccine and vaccine eras. This pioneering study found genotype mixing across countries at shallow phylogenetic depth, revealing frequent long-distance spread of *B. pertussis* isolates and underlining the importance of defining standard genotyping methods that would allow tracing international transmission. Core genome MLST (cgMLST), using the set of genes conserved among isolates of a given bacterial group, represents an approach that combines the high resolution of genome-level variation and the high reproducibility and portability of MLST (*14*). cgMLST genotyping strategies were recently implemented for international coordinated surveillance of several pathogenic bacterial species (*15*–*20*).

We report on the development and evaluation of a cgMLST scheme for genotyping of *B. pertussis* clinical isolates. We demonstrate the resolution power of this approach to recognize groups of intrafamilial isolates or multiple isolates recovered from the same patient. We also show that, in some cases, temporally or geographically unrelated isolates can be nearly undistinguishable, illustrating the rapid diffusion of isolates through hidden chains of transmission. We made the cgMLST strategy for *B. pertussis* isolate characterization publicly available through a Web-accessible genotyping platform (http://bigsdb.pasteur.fr/bordetella), providing a novel tool for tracking the international spread of *B. pertussis* variants.

## Materials and Methods

### Isolates and DNA Preparation

We sequenced a set of 55 isolates (Technical Appendix 1 Table 1). Of these, 24 isolates corresponded to 11 related groups of isolates: 8 isolates originated from 4 different pairs of intrafamilial transmission cases and 16 isolates corresponded to multiple isolates collected from 7 patients (6 pairs and 1 quadruplet); 30 corresponded to a random selection of temporally cocirculating isolates. We used as reference the Tohama isolate (GenBank accession no. NC_002929).

We grew isolates at 36°C for 72 hours on Bordet-Gengou agar (Becton Dickinson, Le Pont de Claix, France) supplemented with 15% defibrinated horse blood (BioMérieux, Marcy l’Étoile, France) and subcultured them in the same medium for 24 hours. We suspended the bacteria in physiologic salt to reach an optical density at 650 nm of 1, and pelleted 400 μL. We suspended the pellets in 100 μL of 1× phosphate-buffered saline, 100 μL of lysis buffer (Roche), and 40 μL of proteinase K; heated them at 65°C for 10 minutes and then at 95°C for 10 minutes; and used them for DNA extraction.

### PFGE 

We obtained PFGE profiles using the *XbaI* enzyme, as described previously (*9*,*21*). We conduated analyses by using BioNumerics version 6.6 (Applied-Maths, Sint-Martens-Latem, Belgium).

### MLVA Analysis

We identified variable-number tandem-repeat (VNTR) sequences (*22*) on each whole-genome sequence using blastn (https://blast.ncbi.nlm.nih.gov/Blast) with Tohama alleles as query. To define Tohama alleles, we located the loci using the primer sequences defined for each locus (VNTR-1, -3, -4, -5, and -6) in the Protocols and Tables section of the Netherlands’ National Institute for Public Health and the Environment’s MLVA website (http://www.mlva.net/bpertussis/default.asp). We defined alleles by counting the number of repeats in the retrieved sequences. We then determined MLVA types using the Single Profile Query section at the same website. 

### WGS, Definition of the Core Genome, and Data Analysis

We describe WGS, our definition of the core genome, and data analysis in Technical Appendix 1. The study accession number in the European Nucleotide Archive is PRJEB21744, including samples ERS1869830–ERS1869884 and their corresponding sequence data.

## Results

### Constitution of the cgMLST Scheme

**Figure 1:**
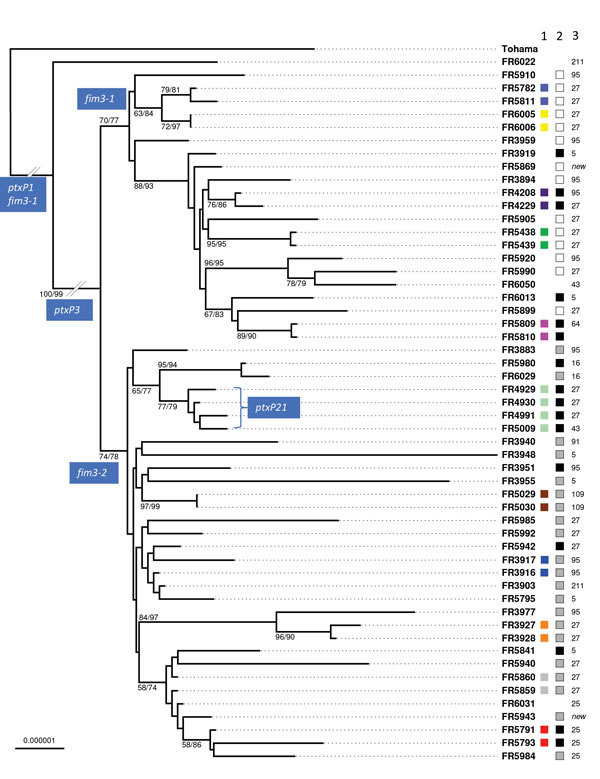
Maximum-likelihood phylogenetic tree for *Bordetella pertussis* isolates based on the concatenated multiple sequence alignments of 2,038 core genome multilocus sequence typing loci. The tree was rooted on the Tohama reference isolate (GenBank accession no. NC_002929). Only branch support values >50 are labeled (bootstrap/aLRT-SH). Column 1 to the right of the isolates’ names shows colors indicating the intrafamilial groups or groups of multiple isolates from single patients (corresponding to colors shown in inner circle of Figure 2). Column 2 shows pulsed-field gel electrophoresis types (French nomenclature; white, IVα; gray, IVβ; black, IVγ). Column 3 shows multilocus variable-number tandem-repeat analysis types. Scale bar indicates nucleotide substitutions per site.

**Figure 2:**
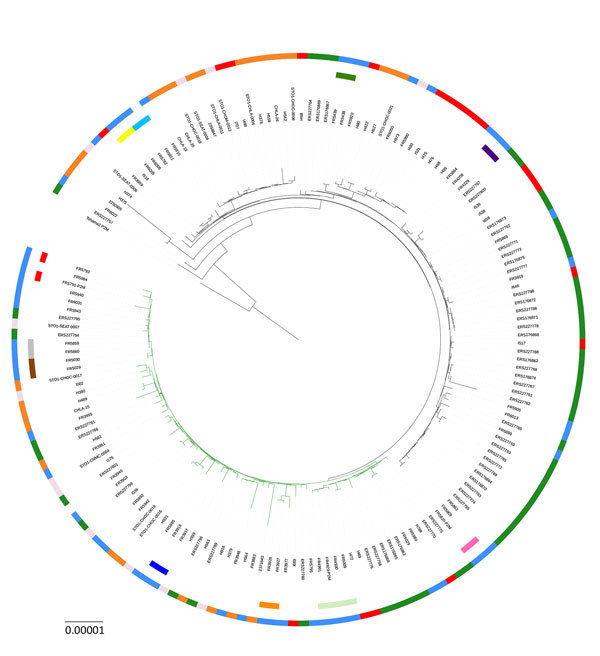
Maximum-likelihood phylogenetic tree for *Bordetella pertussis* based on the concatenated alignments of the 2,038 cgMLST loci sequences of isolates from France (this study) and isolates from outbreaks in the United States and the United Kingdom. The tree was rooted on the Tohama reference isolate (GenBank accession no. NC_002929). Black tree branches indicate *fim3-1* clade and green tree branches indicate *fim3-2* clade. Intrafamilial groups of isolates and multiple groups of isolates recovered from the same patient are represented by sectors of the internal circle surrounding the tree (corresponding to colors in column 1 of Figure 1). The external circle indicates the geographic origin of isolates (blue, France; red, Vermont, USA; orange, California, USA; light pink, other United States; green, United Kingdom). Scale bar indicates nucleotide substitutions per site.

We identified protein-coding genes of *B. pertussis* that were found in >95% of a set of 300 genomes of *B. pertussis* gathered from publicly available data and from our sequencing of isolates from France. We subjected these genes to several filters designed to ensure robustness of genotyping data (Technical Appendix 1). We then chased artifactual variation of allele calls using assemblies available for 3 reference strains obtained from different sequencing methods and assemblies of 17 isolates from France sequenced with different Illumina (San Diego, CA, USA) sequencing systems (HiSeq and NextSeq; Technical Appendix 1 Table 2). We also assessed the dependency of allele calls to assembly coverage depth, by using randomly selected read pairs from raw sequencing data of 1 isolate (FR6072), and eliminated the loci that showed variation above 20× coverage depth. These steps led to a final set of 2,038 gene loci, together constituting a *B. pertussis* cgMLST scheme that should minimize artifactual variation caused by the use of different sequencing platforms or sequencing depths. The set of 2,038 core genes had a total length of 1,751,253 bp, covering 42.9% of the Tohama reference genome. The median gene length was ≈1,000 bp (Technical Appendix 1 Figure 1). Eleven loci were >3,000 bp long and corresponded to genes encoding large proteins such as BrkA, DnaE, RpoB or CyaA (Technical Appendix 2). Most core genes had <10 alleles within our selection of 300 *B. pertussis* genomes used to define the cgMLST scheme, consistent with previous estimates of sequence variation within this homogeneous pathogenic species (*11*,*13*). Core genes belonged to diverse gene classification categories (Technical Appendix 1 Figure 2).

### Phylogenetic Analysis of cgMLST

Phylogenetic analysis of the 55 isolates of the study based on concatenated alignments of the 2,038 gene sequences showed 2 early diverging branches comprising the reference strain Tohama, which belongs to the early-diverging *ptxP1* clade (*13*), and *ptxP1* strain FR6022 (Figure 1). Of the clinical isolates, 49 belonged to clade *ptxP3* and 4 belonged to the previously described clade *ptxP21,* which is derived from *ptxP3* (*23*) (Technical Appendix 1 Table 1). These 53 non-*ptxP1* isolates were separated according to their *fim3* allele, either *fim3-1* or *fim3-2*. These results are congruent with previous phylogenetic analyses (*13*). Cluster analysis of the cgMLST allelic profiles led to a very similar grouping of isolates (Technical Appendix 1 Figure 3), indicating that this method can be used for rapid classification purposes.

Groups of intrafamilial or of multiple isolates from the same patient were largely distributed across the phylogenetic tree (Figure 1). Although most groups formed a distinct branch (Figure 1), 3 sets of isolates (FR3916 and FR3917, FR5859 and FR5860, and FR5791 and FR5793), were intermixed in the tree with isolates with no documented epidemiologic link.

### Numbers of Allelic Differences among Epidemiologically Related or Nonrelated Isolates

When considering the 55 isolates of the study, we found only 1 or 2 uncalled allele(s) among the 2,038 loci of the cgMLST scheme: 53.6% of the isolates had 2,038 core genes tagged, 42.8% had 1 missing allele, and 3 (5.4%) isolates had 2 missing alleles. We evaluated the pairwise comparisons of allelic profiles and recorded the number of mismatches, defined as allelic differences at loci where both isolates had an allele called. We found the highest numbers of allelic mismatches (close to 66 allelic differences) for the comparisons of non-*PtxP1* isolates with the Tohama reference strain, consistent with this strain belonging to a distant lineage. In turn, comparisons of the *ptxP1* isolate FR6022 with the non-*ptxP1* clinical isolates showed 15–25 allelic differences. Among non-*ptxP1* clinical isolates, the number of allelic mismatches varied from 0 to 15 (median 9). All pairwise comparisons between pairs of intrafamilial isolates or pairs of multiple isolates collected from the same patient showed a median value of 1 allelic mismatch (maximum 2). However, 2.8% (39 of 1,415) pairs of nonrelated isolates also showed either 1 or 2 mismatches only.

### Comparison of cgMLST with PFGE and MLVA

Based on PFGE, all clinical isolates belonged to PFGE group IV. This group is highly predominant in France among contemporaneous (post-2000) isolates, and is subdivided into 3 different subgroups, IVα, IVβ, and IVγ (*24*,*25*), which were all represented in our selection. Analysis of the distribution of PFGE profiles along the cgMLST-based phylogenetic tree (Figure 1) revealed that subgroups IVα and IVβ were separated into 2 clades, corresponding with *fim3-1* (associated with IVα) and *fim3-2* (associated with IVβ). In contrast, PFGE subgroup IVγ was found interspersed in these 2 clades, indicating that it does not represent a natural (monophyletic) grouping of *B. pertussis* isolates. As expected, the same PFGE subgroups were shared by nonrelated and related isolates (Figure 1).

We extracted MLVA profiles from whole-genome sequence assemblies. The main MLVA types were MLVA-27 (38.9%), MLVA-95 (18.5%), MLVA-5 (11.1%), and MLVA-25 (7.4%). The first 3 genotypes were distributed widely across the phylogenetic tree (Figure 1), indicating that, similar to PFGE subgroups, they do not represent proper phylogenetic clades.

### Comparison of cgMLST with a Whole-Genome Single Nucleotide Polymorphism–Based Approach

We used a mapping approach against the Tohama strain genome as reference (GenBank accession no. NC_002929), and compared the derived single-nucleotide polymorphism (SNP)–based phylogenetic tree to the one inferred from the concatenated multiple sequence alignments obtained from the cgMLST loci (Technical Appendix 1 Figure 4). Both approaches were highly congruent, grouping the isolates in nearly identical clades. The SNP-based approach led to the identification of 721 variable positions. The highest numbers of SNPs were found for the comparisons of recent isolates (*ptxP3* and *ptxP21*) with the Tohama reference strain (266 ± 10 SNPs) and with the *ptxP1* isolate FR6022 (98 ± 5 SNPs). Among unrelated *ptxP3* clinical isolates, the number of SNPs was 34 ± 9. In the cgMLST gene sequences, there were 206 variable positions, 83.5% of which were included in those identified in the SNP-based analysis. The genome-wide SNP approach might thus be useful as a complementary approach when very high resolution is needed. Comparisons among the 11 related cases showed a very low number of SNPs (no SNP in 9 out of 11 comparisons, 1 SNP in 1 comparison, and 2 SNPs in the remaining comparison), consistent with the cgMLST results. When considering the 3 pairs of isolates not fully resolved using cgMLST, we noticed that FR3916 and FR3917 displayed no SNP between each other, and no SNP with cocirculating isolate FR3903; FR5859 and FR5860 displayed no SNP between each other but >18 SNPs compared with FR5940 and 12 SNPs compared with FR5841; and FR5791 and FR5793 displayed no SNP between each other but 3 or 4 SNPs with cocirculating isolate FR5984. These observations show that, except for the first case, whole-genome SNPs discriminate the related pairs from epidemiologically nonrelated isolates better than cgMLST does. Altogether, these results emphasize that, for the highly monomorphic *B. pertussis*, genotyping data will need to be complemented with epidemiologic data to unravel transmission chains.

### Application of cgMLST to Study of Outbreaks from Different Countries

We analyzed publicly available whole-genome sequences corresponding to 3 outbreaks that occurred in California and Vermont (*26*,*27*), USA, and in the United Kingdom (*4*) (Technical Appendix 1 Table 3). Figure 2 illustrates the phylogenetic relationships of these isolates compared with those from France, based on cgMLST gene sequences. We observed that all pairs of intrafamilial isolates from France and all pairs of multiple isolates recovered from the same patient remained grouped. Isolates from each of the 3 US and UK outbreaks were found in different branches of the phylogenetic tree, consistent with previous results showing that they did not result from the spread of a unique strain (*4*). This finding confirms that the outbreaks of pertussis disease we analyzed corresponded to the simultaneous emergence of multiple strains, consistent with the hypothesis of the silent maintenance of a genetically heterogeneous pool of *B. pertussis* strains in the human population (*4*,*27*).

## Discussion

We developed a cgMLST scheme for *B. pertussis*, one of the most monomorphic bacterial pathogens (*11*,*13*,*28*). Resolving groups of related isolates (such as intrafamilial cases or multiple isolates recovered from the same patient) from nonrelated cocirculating isolates is challenging, given that the *B. pertussis* population is very homogeneous. Consistent with expectations, few of the 2,038 gene loci of the genotyping scheme were variable among non-*ptxP1* isolates. Despite this low level of genetic diversity, the cgMLST scheme grouped most sets of isolates from direct transmission chains and distinguished them most of the time from cocirculating unrelated isolates. Therefore, this genotyping approach will help define chains of transmission of *B. pertussis*. Nevertheless, close genetic relatedness does not demonstrate direct epidemiologic relatedness. Conversely, isolates from different outbreaks (as defined by the notable increase of clinical cases in defined geographic areas) were genetically heterogeneous, demonstrating a diversity of isolates circulating during pertussis epidemics.

Given our ambition to develop a universally applicable cgMLST *B. pertussis* genotyping scheme, we selected the core genes using 300 *B. pertussis* genomes of international origins. Although we cannot exclude that some sublineages of *B. pertussis*, not represented among the 300 isolates, may have lost several of the core loci selected here, we regard this possibility as unlikely and believe that most cgMLST gene loci selected here will be present in most *B. pertussis* isolates.

Remarkably, cgMLST was much more discriminant than PFGE or MLVA, 2 reference epidemiologic typing methods, which appeared unable to distinguish related intrafamilial isolates or isolates collected from the same patient from cocirculating unrelated ones. PFGE and MLVA are widely used and will likely continue to be used until WGS is largely implemented (*29*–*32*), especially in settings in which WGS cannot be achieved because of cost considerations. Our work provides a correspondence between whole-genome–based phylogenetic data and both PFGE and MLVA genotypes and thus provides essential information for the accurate interpretation of typing data from these legacy typing methods. 

Even though standardization efforts have been made, it remains difficult to compare PFGE data from different countries. Using isolates from France and the French nomenclature for PFGE profiles (*9*), we demonstrated that some PFGE subtypes, such as type IV-γ, conflate phylogenetically distinct *B. pertussis* sublineages. MLVA typing is more comparable across laboratories but even less discriminatory than PFGE. In contrast to these 2 legacy typing methods, the phylogenetic tree based on cgMLST genes was highly congruent with that obtained from whole-genome SNPs. Therefore, the cgMLST loci, used in combination, represent powerful phylogenetic markers and will enable identification of meaningful groupings of *B. pertussis* isolates. The cgMLST scheme developed here may therefore be a powerful approach for identifying emerging *B. pertussis* sublineages. Because the cgMLST scheme covers only 43% of the genome, a complementary full-genome analysis will be required to define the particular biologic features, such as the loss of vaccine antigen expression, of emerging sublineages.

The cgMLST scheme developed here forms the basis of a unified allele nomenclature database, which was made openly accessible online at http://bigsdb.pasteur.fr/bordetella. This novel gene-by-gene genotyping strategy (*14*) opens the prospects of an internationally unified surveillance, whereby emerging genotypes and sublineages can be recognized in real time by surveillance laboratories. The ability of different national reference centers, microbiology laboratories, and public health agencies to compare *B. pertussis* genotypes will facilitate understanding of transmission dynamics. Further, the harmonization of epidemiologic typing practice by the use of the same genotyping approach will facilitate sharing of experiences among national surveillance systems and has the potential to promote collaboration. Finally, phylogenetic comparisons of isolates from different countries and world regions will facilitate the much-needed studies of the impact of whole-cell or acellular vaccines and the various vaccination strategies in use (*33*,*34*) on the transmission success of particular *B. pertussis* lineages, such as those that evolve toward a lack of expression of vaccine antigens.

Technical Appendix 1 Description of *Bordetella pertussis* whole-genome sequencing and analysis processes.

Technical Appendix 2 *Bordetella pertussis* core gene characteristics.
