# Supplementary material for: Genomic Sequencing of Bordetella pertussis for Epidemiology and Global Surveillance of Whooping Cough
Source: Emerg Infect Dis. 2018 Jun;24(6):988–94. doi: 10.3201/eid2406.171464 (PMC6004856; doi:10.3201/eid2406.171464)
Supplement: Technical Appendix 1 — Description of Bordetella pertussis whole-genome sequencing and analysis processes. [file 17-1464-Techapp-s1.pdf]

Article DOI: https://doi.org/10.3201/eid2406.171464

Genomic Sequencing of *Bordetella pertussis* for Epidemiology and Global Surveillance of Whooping Cough

Technical Appendix 1

Whole-Genome Sequencing

We constructed libraries using the Nextera XT DNA Library Preparation kit (Illumina, San Diego, CA). We performed WGS on a NextSeq 500 system (Illumina) using a 2×150 paired-end protocol at the Mutualized Platform for Microbiology of Institut Pasteur.

De Novo Genome Assembly

For de novo assembly, paired-end reads were clipped and trimmed with AlienTrimmer (*1*), corrected with Musket (*2*), merged (if needed) with FLASH (*3*), and subjected to a digital normalization procedure with khmer (*4*). For each sample, remaining processed reads were assembled and scaffolded with SPAdes (*5*).

Definition of Core Genome

We selected 300 genomes of *Bordetella pertussis*. Of these, 29% were downloaded from NCBI (https://www.ncbi.nlm.nih.gov/genome/genomes/1008; Technical Appendix 1 Table 4); the others were genomes of French isolates selected according to the following criteria: all isolates collected in 2015 and 2016 at the date of study design; a random selection of 10 isolates per year from 2004 to 2014; and a representative selection of at least 5 vaccine antigen-deficient isolates per year and as many antigen producing isolates in the same year.

From this set of 300 genomes, we inferred the species core genome using the CoreGeneBuilder pipeline (https://zenodo.org/record/165206#.WVpT7I55EQU) with *B. pertussis* strain Tohama I (GenBank accession no. NC_002929) as reference. CoreGeneBuilder automatically removed genomes for which the size was too divergent compared with the whole

set. We applied the following quality filters: a maximum of 500 contigs and a minimum N50 of 10,000 bp. With those criteria, no genome was filtered out.

The pipeline's next step relies on the eCAMBer software (*6*), which consists of a de novo annotation of the genomes (except the reference) using Prodigal (*7*) and in the harmonization of the positions of the stop and start codons. In the last step, the core genome is inferred using a bidirectional best hits approach, following Touchon et al. (*8*). We used CoreGeneBuilder default settings, and considered a gene as part of the core genome if it was found in at least 95% of the 300 selected genomes. This resulted in an initial core genome containing 3,125 loci.

Core Gene Filtering

To obtain a set of loci that would be highly robust to genotyping artifacts, we filtered out some genes based on several criteria.

First, we removed potential paralogs. The presence of paralogs inside a typing scheme can lead to ambiguities, because a candidate gene might be attributable to 2 different core gene loci. To detect those potential paralogs, we compared each allele of each locus against all the alleles of all the other loci using the software BLAT (*9*). If a single hit was found between 2 different loci (more than 70% amino acid sequence identity between two alleles), we removed both. A total of 187 loci were discarded that way.

We also decided to remove genes that belong to the MLST scheme (*10*) and the ribosomal genes that are used in the ribosomal MLST approach (*11*), so that they could be analyzed independently.

We also removed loci whose length varies too much among alleles, which is useful to reduce ambiguities during the genotyping process. We therefore aligned the amino acid sequences and removed those for which the alignment contained more than 10% of gaps (total number of gaps compared with the total number of character states). This concerned 903 loci.

Allele calling relies on BLASTn (*12*), which does not produce full-length alignments when the start or stop codons differ. We thus decided to remove all loci with variation within the start and stop codons. We discarded 363 loci this way. In addition, we removed 1 locus because at least 1 allele showed 1 or more ambiguous character state(s).

Finally, to avoid redundancy in the information contained within the cgMLST scheme, we removed 60 loci that were overlapping others in the reference genome. We used the definitions of Prodigal for the overlapping genes: a minimum of 60 bp of overlap if genes are on the same strand, and 200 bp if genes are on different strands. A single locus might correspond to several of those filtering criteria. The total number of removed loci was 1,020. Therefore, 2,105 loci were retained at this stage.

The reproducibility of allele calling results at each locus of the core genome was checked by proceeding to pairwise allelic comparisons from several whole-genome assemblies. We first compared assemblies available for each of 3 reference strains (Tohama I, Cs, and 18323), which were obtained from different sequencing methods (online Technical Appendix 1 Table 2). We next compared allele calls for 17 pairwise comparisons for French isolates sequenced with different Illumina sequencing systems (HiSeq and NextSeq). WGS using the HiSeq 2000 instrument (Illumina) was performed at the Institut Pasteur Genomics Platform. In addition, we tested the robustness of allele calling to coverage depth. For that purpose, we used a random selection of reads representing 10 to 50× coverage depths by selecting, after quality preprocessing of the reads, a random subsample of reads obtained from the FR6072 isolate using an Illumina NextSeq 500 sequencing system (corresponding to an initial 94× coverage depth). For each of the simulated coverage depth levels, we evaluated the reproducibility of allele calling using 10 distinct genome assemblies. We detected 67 loci that showed variation of allele calls with these tests. Most were variable within multiple comparisons of assemblies derived from single isolates. Visual inspection of coverage depth at these loci by using a read mapping approach revealed a local drop in sequencing depth, attributable in many cases to a very high G+C content of 1 or more sequence region(s) of the loci. All loci with variation in at least 1 pair from these reproducibility tests were discarded.

We thereby obtained a final set of 2,038 core genes, which together constitute the *B. pertussis* cgMLST scheme that was evaluated in this study.

Phylogenetic Analysis

To derive a phylogenetic tree based on cgMLST loci, we extracted the amino acid allele sequences of each locus and aligned them with MAFFT v7 (*13*). We then back-translated multiple amino acid sequence alignments to codon alignments; a concatenation of all loci yielded

a supermatrix of 1,751,253 nt characters. We used IQ-TREE v1.5.4 (*14*) to infer a phylogenetic tree from this supermatrix of characters with a GTR+Γ_4+I evolutionary model. We assessed branch supports with both bootstrap (1,000 replicates) and aLRT-SH methods (*15*).

SNP-Based Analysis

We selected the assembled genome of the strain Tohama I (GenBank accession no. NC_002929) as a reference to perform read mapping of each of the sequenced samples with BWA (*16*). For each sample, we defined each nucleotide base of the reference as undercovered if the number of aligned reads was lower than the 25th or or overcovered if the number of aligned reads was higher than the 975th permille of the overall read coverage distribution. For each sample, read alignments were used to infer a pseudo-genome with the following 3 rules:

1) Each position is replaced by the degenerated character state N if every nucleotide base occurs in <80% of the covering character states at that position, or otherwise by the most frequently occurring character state, which can be ACGT or a gap, denoted by a dash (–).

2) Every undercovered position is replaced by the unknown character state ?.

3) Every SNP position (i.e., the reference and inferred character states are different) located within a region that is overcovered or strand-biased (i.e., <5 aligned reads on at least 1 strand) was replaced by the character state X.

Following this approach, we obtained pseudo-genome sequences with identical lengths, leading to a multiple sequence alignment. After removing every position containing >25% undefined character states (–, N, X and ?), we used the remaining characters to infer a maximum likelihood phylogenetic tree with IQ-TREE (*14*) and the same evolutionary model as for the cgMLST gene loci concatenated (as discussed previously).

Recommendation for Querying cgMLST Alleles Inside a Genome

When using de novo assembly of Illumina reads, we recommend a coverage depth of at least 40× after preprocessing (e.g., quality trimming) of reads to define alleles accurately.

References

1. Criscuolo A, Brisse S. AlienTrimmer: a tool to quickly and accurately trim off multiple short contaminant sequences from high-throughput sequencing reads. Genomics. 2013;102:500–6. http://dx.doi.org/10.1016/j.ygeno.2013.07.011

2. Liu Y, Schröder J, Schmidt B. Musket: a multistage *k*-mer spectrum-based error corrector for Illumina sequence data. Bioinformatics. 2013;29:308–15. http://dx.doi.org/10.1093/bioinformatics/bts690

3. Magoč T, Salzberg SL. FLASH: fast length adjustment of short reads to improve genome assemblies. Bioinformatics. 2011;27:2957–63. http://dx.doi.org/10.1093/bioinformatics/btr507

4. Crusoe MR, Alameldin HF, Awad S, Boucher E, Caldwell A, Cartwright R, et al. The khmer software package: enabling efficient nucleotide sequence analysis. F1000Res. 2015;4:900. http://dx.doi.org/10.12688/f1000research.6924.1

5. Bankevich A, Nurk S, Antipov D, Gurevich AA, Dvorkin M, Kulikov AS, et al. SPAdes: a new genome assembly algorithm and its applications to single-cell sequencing. J Comput Biol. 2012;19:455–77. http://dx.doi.org/10.1089/cmb.2012.0021

6. Wozniak M, Tiuryn J, Wong L. GWAMAR: genome-wide assessment of mutations associated with drug resistance in bacteria. BMC Genomics. 2014;15(Suppl 10):S10. http://dx.doi.org/10.1186/1471-2164-15-S10-S10

7. Hyatt D, Chen GL, Locascio PF, Land ML, Larimer FW, Hauser LJ. Prodigal: prokaryotic gene recognition and translation initiation site identification. BMC Bioinformatics. 2010;11:119. http://dx.doi.org/10.1186/1471-2105-11-119

8. Touchon M, Hoede C, Tenaillon O, Barbe V, Baeriswyl S, Bidet P, et al. Organised genome dynamics in the *Escherichia coli* species results in highly diverse adaptive paths. PLoS Genet. 2009;5:e1000344. http://dx.doi.org/10.1371/journal.pgen.1000344

9. Kent WJ. BLAT—the BLAST-Like Alignment Tool. Genome Res. 2002;12:656–64. http://dx.doi.org/10.1101/gr.229202

10. Diavatopoulos DA, Cummings CA, Schouls LM, Brinig MM, Relman DA, Mooi FR. *Bordetella pertussis*, the causative agent of whooping cough, evolved from a distinct, human-associated lineage of *B. bronchiseptica*. PLoS Pathog. 2005;1:e45. http://dx.doi.org/10.1371/journal.ppat.0010045

11. Jolley KA, Bliss CM, Bennett JS, Bratcher HB, Brehony C, Colles FM, et al. Ribosomal multilocus sequence typing: universal characterization of bacteria from domain to strain. Microbiology. 2012;158:1005–15. http://dx.doi.org/10.1099/mic.0.055459-0

12. Altschul SF, Gish W, Miller W, Myers EW, Lipman DJ. Basic local alignment search tool. J Mol Biol. 1990;215:403–10. http://dx.doi.org/10.1016/S0022-2836(05)80360-2

13. Katoh K, Standley DM. MAFFT multiple sequence alignment software version 7: improvements in performance and usability. Mol Biol Evol. 2013;30:772–80. http://dx.doi.org/10.1093/molbev/mst010

14. Nguyen LT, Schmidt HA, von Haeseler A, Minh BQ. IQ-TREE: a fast and effective stochastic algorithm for estimating maximum-likelihood phylogenies. Mol Biol Evol. 2015;32:268–74. http://dx.doi.org/10.1093/molbev/msu300

15. Anisimova M, Gil M, Dufayard JF, Dessimoz C, Gascuel O. Survey of branch support methods demonstrates accuracy, power, and robustness of fast likelihood-based approximation schemes. Syst Biol. 2011;60:685–99. http://dx.doi.org/10.1093/sysbio/syr041

16. Li H. Aligning sequence reads, clone sequences and assembly contigs with BWA-MEM. arXiv:1303.3997;2013.

17. Bart MJ, Harris SR, Advani A, Arakawa Y, Bottero D, Bouchez V, et al. Global population structure and evolution of *Bordetella pertussis* and their relationship with vaccination. MBio. 2014;5:e01074-14. http://dx.doi.org/10.1128/mBio.01074-14

18. Park J, Zhang Y, Buboltz AM, Zhang X, Schuster SC, Ahuja U, et al. Comparative genomics of the classical *Bordetella* subspecies: the evolution and exchange of virulence-associated diversity amongst closely related pathogens. BMC Genomics. 2012;13:545. http://dx.doi.org/10.1186/1471-2164-13-545

19. Bowden KE, Weigand MR, Peng Y, Cassiday PK, Sammons S, Knipe K, et al. Genome structural diversity among 31 *Bordetella pertussis* isolates from two recent U.S. whooping cough statewide epidemics. mSphere. 2016;1(3).

20. Zhang S, Xu Y, Zhou Z, Wang S, Yang R, Wang J, et al. Complete genome sequence of *Bordetella pertussis* CS, a Chinese pertussis vaccine strain. J Bacteriol. 2011;193:4017–8. http://dx.doi.org/10.1128/JB.05184-11

21. Hegerle N, Paris AS, Brun D, Dore G, Njamkepo E, Guillot S, et al. Evolution of French *Bordetella pertussis* and *Bordetella parapertussis* isolates: increase of Bordetellae not expressing pertactin. Clin Microbiol Infect. 2012;18:E340–6. http://dx.doi.org/10.1111/j.1469-0691.2012.03925.x

22. Bouchez V, Brun D, Cantinelli T, Dore G, Njamkepo E, Guiso N. First report and detailed characterization of *B. pertussis* isolates not expressing pertussis toxin or pertactin. Vaccine. 2009;27:6034–41. http://dx.doi.org/10.1016/j.vaccine.2009.07.074

23. Hegerle N, Dore G, Guiso N. Pertactin deficient *Bordetella pertussis* present a better fitness in mice immunized with an acellular pertussis vaccine. Vaccine. 2014;32:6597–600. http://dx.doi.org/10.1016/j.vaccine.2014.09.068

24. Harvill ET, Goodfield LL, Ivanov Y, Meyer JA, Newth C, Cassiday P, et al. Genome sequences of 28 *Bordetella pertussis* U.S. outbreak strains dating from 2010 to 2012. Genome Announc. 2013;1:e01075-13. http://dx.doi.org/10.1128/genomeA.01075-13

25. Sealey KL, Harris SR, Fry NK, Hurst LD, Gorringe AR, Parkhill J, et al. Genomic analysis of isolates from the United Kingdom 2012 pertussis outbreak reveals that vaccine antigen genes are unusually fast evolving. J Infect Dis. 2015;212:294–301. http://dx.doi.org/10.1093/infdis/jiu665

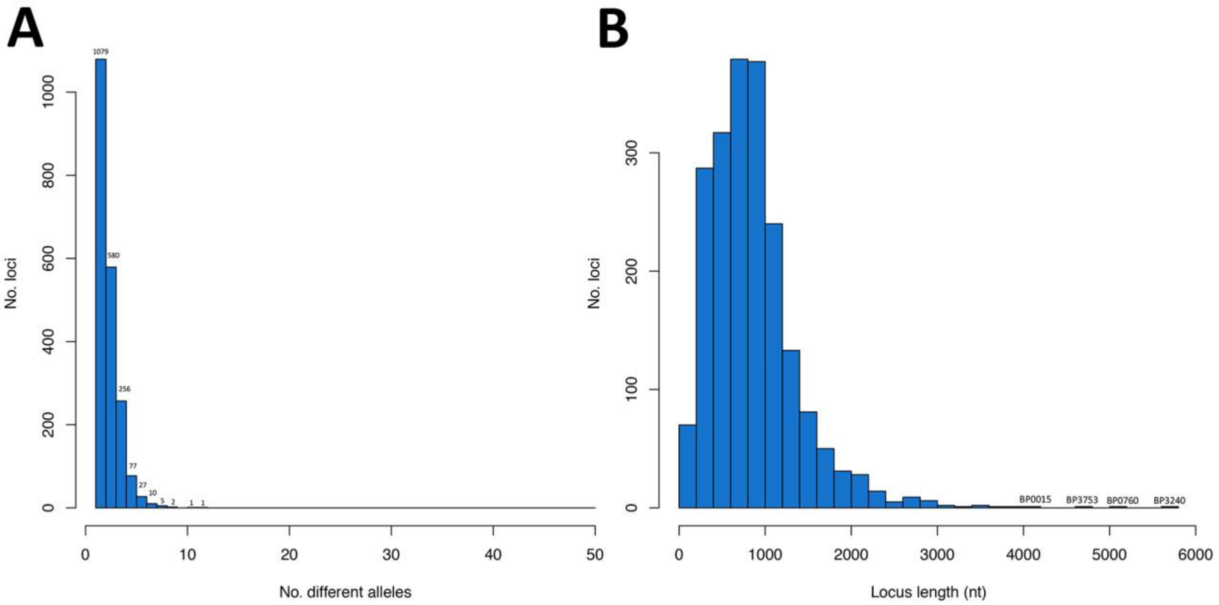

Technical Appendix 1 Figure 1. Distribution of the number of cgMLST loci of *B. pertussis* as a function of the number of distinct alleles (A) and of locus length (B).

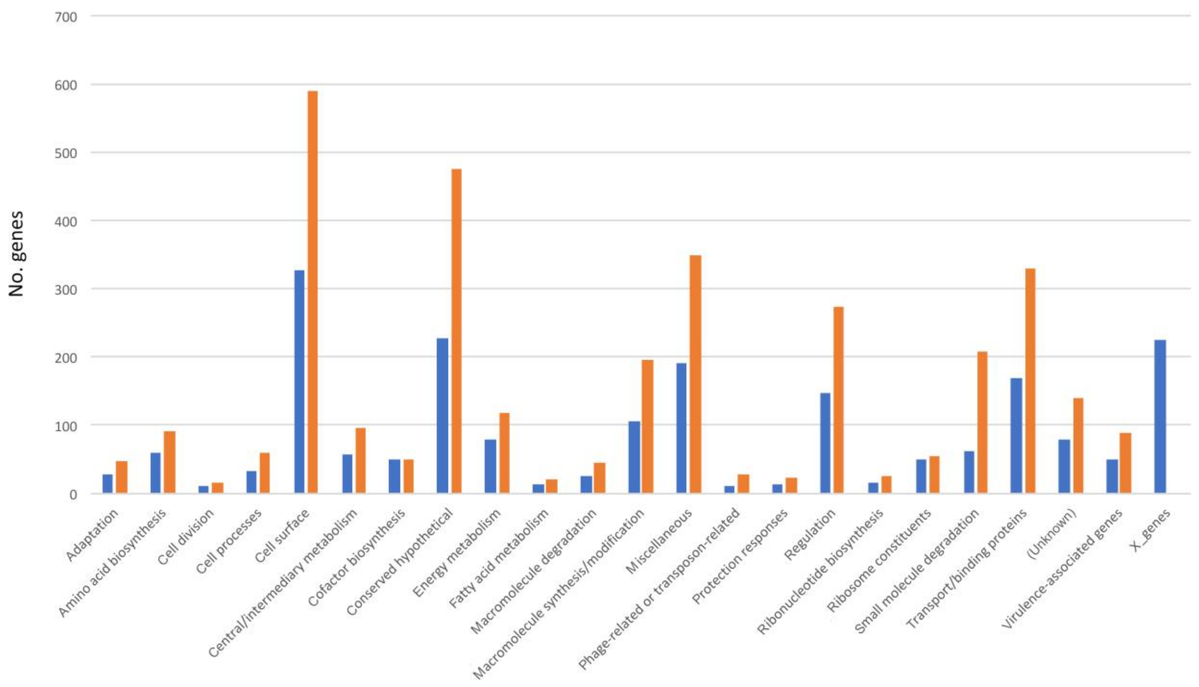

Technical Appendix 1 Figure 2. Repartition of the cgMLST genes of *B. pertussis* into functional categories (in blue), compared with all genes of the Tohama reference strain (in orange). Gene categories were obtained from Bart et al., MBio 2014 (*17*). X_genes correspond to loci that were not annotated as CDSs on the reference strain.

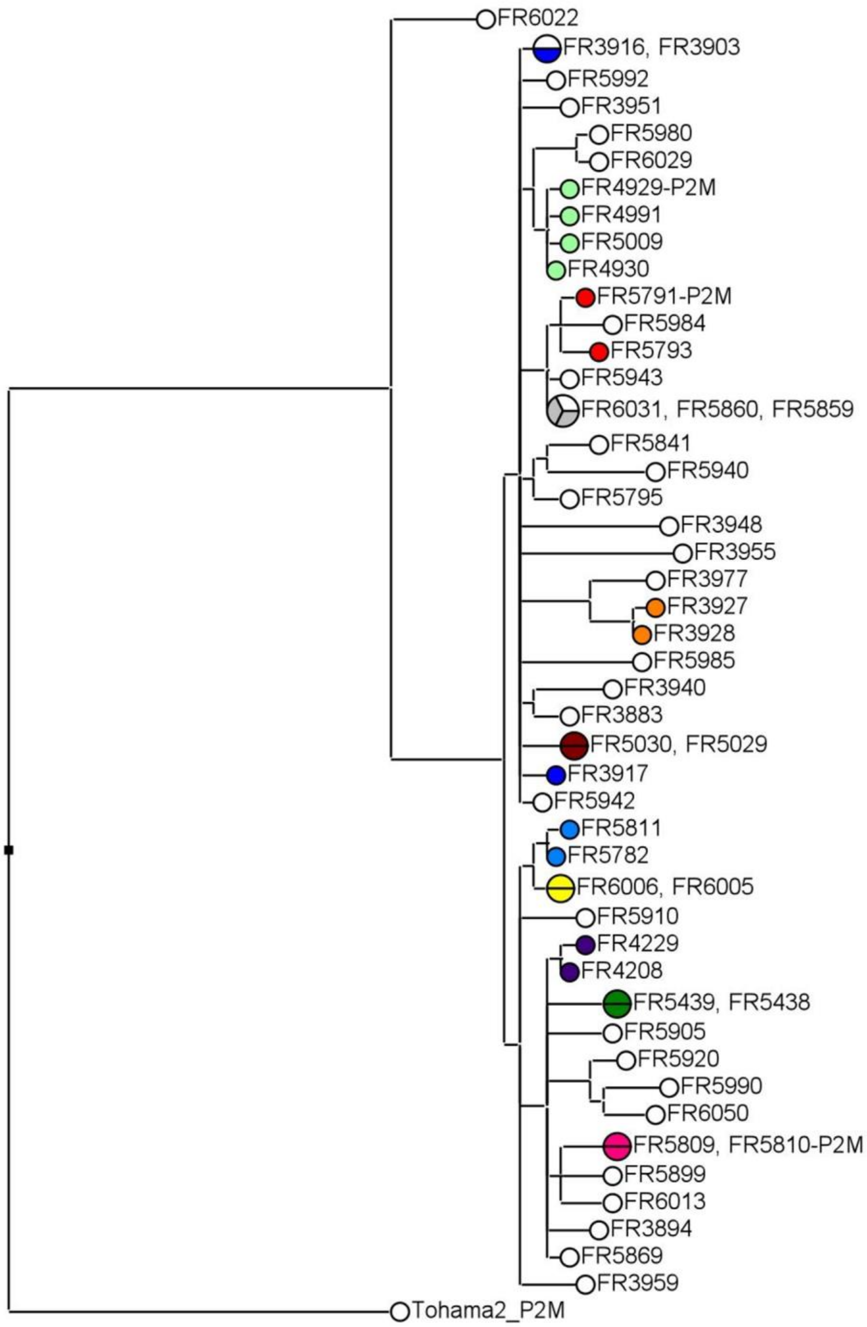

Technical Appendix 1 Figure 3. Maximum parsimony tree rooted on the Tohama strain of *B. pertussis*, based on the matrix of the number of allelic mismatches among cgMLST profiles (obtained using Bionumerics v7.6, Applied-Maths, Sint-Martens-Latem, Belgium). The colors of tree leaves circles indicate intrafamilial groups and groups of multiple isolates from single patients (colors are the same as in Figure 1 in the main article text). Pairwise comparisons leading to no allelic differences were merged in the same circle.

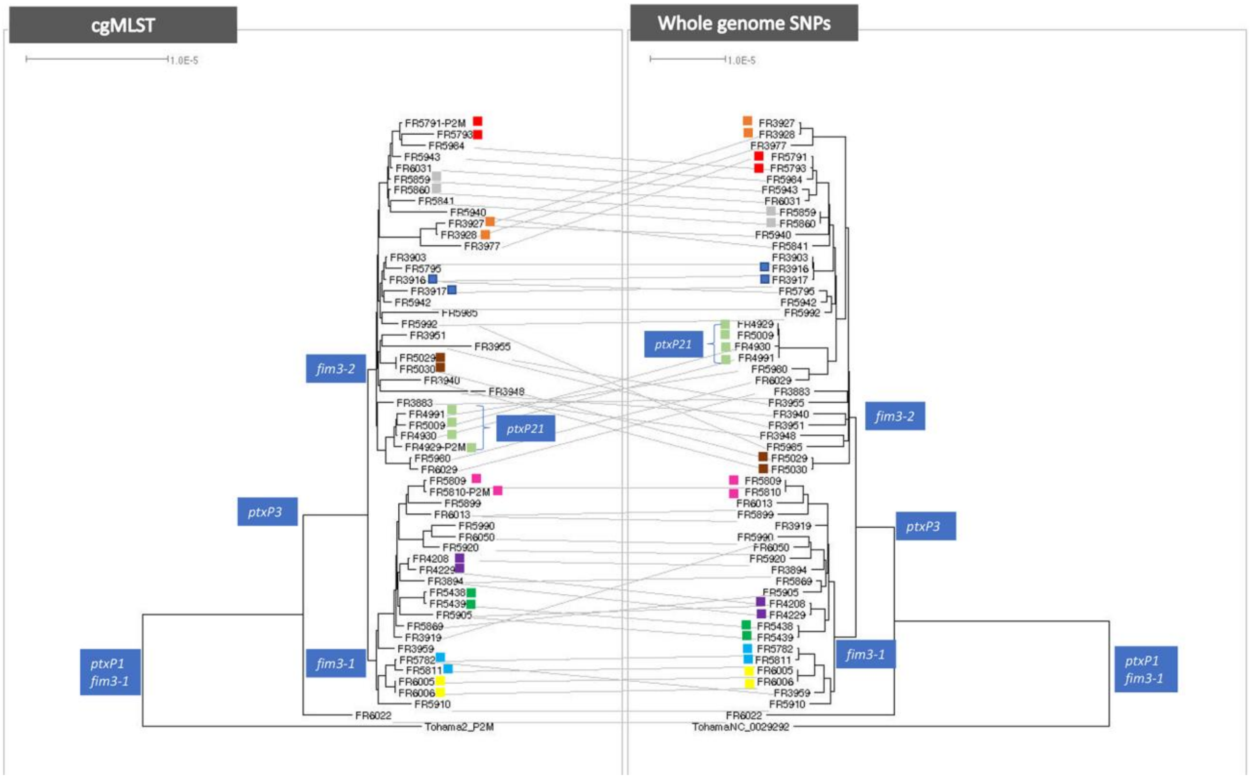

Technical Appendix 1 Figure 4. Comparison of the phylogenetic trees of *B. pertussis* obtained either from the concatenated cgMLST loci (left) or from the read mapping approach (*3*). The figure was obtained with Dendroscope (http://dendroscope.org/) using the tanglegram functionality. The colored squares in front of groups of isolates indicate intrafamilial groups and groups of multiple isolates from single patients (colors are the same as in Figure 1 in the main article text).

Technical Appendix 1 Table 1. Characteristics of *B. pertussis* isolates and their genome assemblies*

Name of isolate	Id_isolate	Country	Type of isolate	Year	PFGE	MLVA	Serotype	ptxP	ptxA	fim2	fim3	PRN	Avg_flt_cov	Total length	No._contigs	N50_contig_length
FR3883	FR3883	France	Co-circulating	2008	IVβ	95	2–/3+	ptxP3	ptxA1	fim2-1	fim3-2	PRN2	87	3860563	295	20090
FR3894	FR3894	France	Co-circulating	2008	IVα	95	2–/3+	ptxP3	ptxA1	fim2-1	fim3-1	PRN2	89	3857564	283	20927
FR3903	FR3903	France	Co-circulating	2008	IVβ	211	2–/3+	ptxP3	ptxA1	fim2-1	fim3-2	PRN2	90	3867534	285	20805
FR3916	FR3916	France	Intrafamilial	2008	IVβ	95	2–/3+	ptxP3	ptxA1	fim2-1	fim3-2	PRN2	85	3858307	291	20751
FR3917	FR3917	France	Intrafamilial	2008	IVβ	95	2–/3+	ptxP3	ptxA1	fim2-1	fim3-2	PRN2	91	3853135	291	20490
FR3919	FR3919	France	Co-circulating	2008	IVγ	5	2–/3+	ptxP3	ptxA1	fim2-1	fim3-1	PRN2	91	3858784	293	20532
FR3927	FR3927	France	Single patient	2008	IVβ	27	2–/3+	ptxP3	ptxA1	fim2-1	fim3-2	PRN2	94	3871790	283	20533
FR3928	FR3928	France	Single patient	2008	IVβ	27	2–/3+	ptxP3	ptxA1	fim2-1	fim3-2	PRN2	94	3853225	285	20490
FR3940	FR3940	France	Co-circulating	2008	IVβ	91	2–/3+	ptxP3	ptxA1	fim2-1	fim3-2	PRN2	74	3869563	304	20107
FR3948	FR3948	France	Co-circulating	2008	IVβ	5	2–/3+	ptxP3	ptxA1	fim2-1	fim3-2	PRN2	60	3861132	303	20086
FR3951	FR3951	France	Co-circulating	2008	IVγ	95	2–/3+	ptxP3	ptxA1	fim2-1	fim3-2	PRN2	87	3857150	284	20927
FR3955	FR3955	France	Co-circulating	2008	IVβ	5	2–/3+	ptxP3	ptxA1	fim2-1	fim3-2	PRN2	84	3850150	294	20334
FR3959	FR3959	France	Co-circulating	2008	IVα	95	2–/3+	ptxP3	ptxA1	fim2-1	fim3-1	PRN2	79	3859301	299	20093
FR3977	FR3977	France	Co-circulating	2008	IVβ	95	2–/3+	ptxP3	ptxA1	fim2-1	fim3-2	PRN2	84	3877182	305	20421
FR4208	FR4208	France	Single patient	2009	IVγ	95	2–/3+	ptxP3	ptxA1	fim2-1	fim3-1	PRN2	95	3865790	283	20790
FR4229	FR4229	France	Single patient	2009	IVγ	27	2–/3+	ptxP3	ptxA1	fim2-1	fim3-1	PRN2	95	3864088	288	20356
FR4929	FR4929-P2M	France	Single patient	2009	IVγ	27	2–/3+	ptxP21	ptxA1	fim2-1	fim3-2	PRN2	96	3874730	286	20827
FR4930	FR4930	France	Single patient	2009	IVγ	27	2–/3+	ptxP21	ptxA1	fim2-1	fim3-2	PRN2	79	3840923	284	20728
FR4991	FR4991	France	Single patient	2009	IVγ	27	2–/3+	ptxP21	ptxA1	fim2-1	fim3-2	PRN2	80	3846706	292	20140
FR5009	FR5009	France	Single patient	2011	IVγ	43	2–/3+	ptxP21	ptxA1	fim2-1	fim3-2	PRN2	79	3846125	293	20504
FR5029	FR5029	France	Single patient	2011	IVβ	109	2–/3+	ptxP3	ptxA1	fim2-1	fim3-2	PRN2	92	3859785	287	20755
FR5030	FR5030	France	Single patient	2011	IVβ	109	2–/3+	ptxP3	ptxA1	fim2-1	fim3-2	PRN2	91	3859996	287	20346
FR5438	FR5438	France	Single patient	2013	IVα	27	2–/3+	ptxP3	ptxA1	fim2-1	fim3-1	PRN15	88	3867428	296	19922
FR5439	FR5439	France	Single patient	2013	IVα	27	2–/3+	ptxP3	ptxA1	fim2-1	fim3-1	PRN15	85	3885947	298	20138
FR5782	FR5782	France	Single patient	2014	IVα	27	2–/3+	ptxP3	ptxA1	fim2-1	fim3-1	PRN2	83	3863866	289	20543
FR5791	FR5791-P2M	France	Intrafamilial	2014	IVγ	25	2–/3+	ptxP3	ptxA1	fim2-1	fim3-2	PRN2	73	3862290	298	19922
FR5793	FR5793	France	Intrafamilial	2014	IVγ	25	2–/3+	ptxP3	ptxA1	fim2-1	fim3-2	PRN2	80	3857762	293	20143
FR5795	FR5795	France	Co-circulating	2014	IVβ	5	2–/3+	ptxP3	ptxA1	fim2-1	fim3-2	PRN2	81	3936522	296	20516
FR5809	FR5809	France	Single patient	2014	IVγ	64	2+/3–	ptxP3	ptxA1	fim2-1	fim3-1	PRN2	84	3855030	298	20099
FR5810	FR5810-P2M	France	Single patient	2014	IVγ	ND	2+/3–	ptxP3	ptxA1	fim2-1	fim3-1	PRN2	91	3860415	290	20354
FR5811	FR5811	France	Single patient	2014	IVα	27	2–/3+	ptxP3	ptxA1	fim2-1	fim3-1	PRN2	85	3867077	297	20346
FR5841	FR5841	France	Co-circulating	2014	IVγ	5	2–/3+	ptxP3	ptxA1	fim2-1	fim3-2	PRN2	88	3885468	290	20497
FR5859	FR5859	France	Intrafamilial	2014	IVβ	27	2–/3+	ptxP3	ptxA1	fim2-1	fim3–2	PRN2	95	3869507	312	19411
FR5860	FR5860	France	Intrafamilial	2014	IVβ	27	2–/3+	ptxP3	ptxA1	fim2-1	fim3-2	PRN2	86	3863579	295	20353
FR5869	FR5869	France	Co-circulating	2014	IVα	New	2–/3+	ptxP3	ptxA1	fim2-1	fim3-1	PRN2, SNP1812	85	3878775	288	20411
FR5899	FR5899	France	Co-circulating	2014	IVα	27	2+/3–	ptxP3	ptxA1	fim2-1	fim3-1	PRN2	83	3867799	291	20562
FR5905	FR5905	France	Co-circulating	2014	IVα	27	2–/3+	ptxP3	ptxA1	fim2-1	fim3-1	Deletion till 1603	64	3859440	297	20755

Name of isolate	Id_isolate	Country	Type of isolate	Year	PFGE	MLVA	Serotype	ptxP	ptxA	fim2	fim3	PRN	Avg_flt_ cov	Total length	No._ contigs	N50_ contig_ length
FR5910	FR5910	France	Co-circulating	2014	IVα	95	2–/3+	ptxP3	ptxA1	fim2-1	fim3-1	Deletion till 1603	82	3842529	287	19850
FR5920	FR5920	France	Co-circulating	2014	IVα	95	2–/3+	ptxP3	ptxA1	fim2-1	fim3-1	PRN2	91	3849490	292	20336
FR5940	FR5940	France	Co-circulating	2014	IVβ	27	2–/3+	ptxP3	ptxA1	fim2-1	fim3-2	PRN2	59	3863832	300	19682
FR5942	FR5942	France	Co-circulating	2014	IVγ	27	2–/3+	ptxP3	ptxA1	fim2-1	fim3-2	PRN2	85	3889660	288	20903
FR5943	FR5943	France	Co-circulating	2014	IV β	New	2–/3+	ptxP3	ptxA1	fim2-1	fim3-2	PRN2	90	3861086	287	20338
FR5980	FR5980	France	Co-circulating	2015	IVγ	16	2–/3+	ptxP3	ptxA1	fim2-1	fim3-2	PRN2	80	3856261	297	20683
FR5984	FR5984	France	Co-circulating	2015	IVβ	25	2–/3+	ptxP3	ptxA1	fim2-1	fim3-2	PRN2	80	3861931	305	20346
FR5985	FR5985	France	Co-circulating	2015	IVβ	27	2+/3–	ptxP3	ptxA1	fim2-1	fim3-2	PRN2	80	3856579	295	20494
FR5990	FR5990	France	Co-circulating	2015	IVα	27	2–/3+	ptxP3	ptxA1	fim2–1	fim3-1	PRN2, IS1613	78	3859870	290	20596
FR5992	FR5992	France	Co-circulating	2015	IVβ	27	2–/3+	ptxP3	ptxA1	fim2–1	fim3–2	PRN2	77	3859556	293	20799
FR6005	FR6005	France	Intrafamilial	2015	IVα	27	2–/3+	ptxP3	ptxA1	fim2–1	fim3–1	PRN2	82	3859942	302	20879
FR6006	FR6006	France	Intrafamilial	2015	IVα	27	2–/3+	ptxP3	ptxA1	fim2–1	fim3–1	PRN2	81	3863715	314	20510
FR6013	FR6013	France	Co-circulating	2015	IVγ	5	2+/3–	ptxP3	ptxA1	fim2–1	fim3–1	PRN2	65	3860466	308	20728
FR6022	FR6022	France	Co-circulating	2015	ND	211	2+/3–	ptxP1	ptxA1	fim2–1	fim3–1	PRN2	77	3930904	304	19926
FR6029	FR6029	France	Co-circulating	2015	IVβ	16	2–/3+	ptxP3	ptxA1	fim2–1	fim3–2	PRN2	77	3902002	287	20544
FR6031	FR6031	France	Co-circulating	2015	IVβ	25	Auto	ptxP3	ptxA1	fim2–1	fim3–2	PRN2	70	3869787	293	20346
FR6050	FR6050	France	Co-circulating	2015	ND	43	2–/3+	ptxP3	ptxA1	fim2–1	fim3–1	PRN2, IS1613	76	3854706	300	20142
Tohama	Tohama2 _P2M	Japan	Reference	1954	II	83	2+/3–	ptxP1	ptxA2	fim2–1	fim3–1	PRN1	98	3875676	279	21069

*MLVA, multilocus variable number of tandem repeats; ND, not defined; PFGE, pulsed-field gel electrophoresis

Technical Appendix 1 Table 2. Characteristics of *B. pertussis* isolates used in reproducibility tests

Name of isolate	Id_isolate	Technology	No. contigs	Assembly length (bp)	GenBank accession no.	Reference
18323	18323	PacBio	4	4135236	ASM152555v1	Unpublished
	18323-2	Sanger	1	4043846	NC_018518.1	(*18*)
	18323-R1*	Illumina NextSeq	285	3826649		This study
	18323-R2*	Illumina NextSeq	283	3852915		This study
Tohama	E476	PacBio/Illumina	1	4102978	CP010964	(*19*)
	Tohama2_P2M*	Illumina-NextSeq	271	3918005		This study
	8132-P2M*	Illumina-NextSeq	279	3875676		This study
Cs	Cs	Roche454+Sanger	1	4124236	NC_017223	(*20*)
	C393	PacBio	1	4133777	CP010963	(*19*)
FR5819	FR5819-P2M*	Illumina-NextSeq	302	3870191		This study
	FR5819-P2M2*	Illumina-NextSeq	292	3861533		This study
FR6072	FR6072	Illumina-NextSeq	279	3868752		This study
	FR6072-R1*	Illumina NextSeq	284	3881279		This study
	FR6072-R2*	Illumina NextSeq	286	3876991		This study
FR0432	FR0432	Illumina HiSeq	278	3912480		This study
	FR432-P2M*	Illumina NextSeq	298	3882826		This study
FR0658	FR0658	Illumina HiSeq	272	3898466		This study
	FR658-P2M*	Illumina NextSeq	287	3872071		This study
FR3469	FR3469	Illumina HiSeq	268	3895018		(*21*)
	FR3469-P2M*	Illumina NextSeq	282	3867615		(*21*)
FR3693	FR3693	Illumina HiSeq	270	3898970		(*22*)
	FR3693-P2M*	Illumina-NextSeq	282	3868130		(*22*)
FR3749	FR3749	Illumina HiSeq	273	3873750		(*22*)
	FR3749-P2M*	Illumina NextSeq	293	3838247		(*22*)
FR5388	FR5388	Illumina HiSeq	273	3865427		(*23*)
	FR5388-P2M*	Illumina NextSeq	284	3885354		(*23*)
FR5392	FR5392	Illumina HiSeq	274	3860575		(*23*)
	FR5392-P2M*	Illumina NextSeq	286	3937575		(*23*)
FR5947	FR5947	Illumina HiSeq	270	3899479		This study
	FR5947-P2M*	Illumina NextSeq	289	3861575		This study
FR5787	FR5787	Illumina HiSeq	274	3930834		This study
	FR5787-P2M*	Illumina NextSeq	294	3898919		This study
FR5791	FR5791	Illumina HiSeq	268	3860976		This study
	FR5791-P2M*	Illumina NextSeq	298	3862290		This study
FR5794	FR5794	Illumina HiSeq	271	3864747		This study
	FR5794-P2M*	Illumina NextSeq	301	3870678		This study
FR5810	FR5810	Illumina HiSeq	273	3864244		This study
	FR5810-P2M*	Illumina-NextSeq	290	3860415		This study
FR5819	FR5819	Illumina HiSeq	272	3864507		This study
	FR5819-P2M*	Illumina NextSeq	302	3870191		This study
	FR5819-P2M2*	Illumina NextSeq	292	3861533		This study
FR5845	FR5845	Illumina HiSeq	270	3862629		This study
	FR5845-P2M*	Illumina NextSeq	285	3864915		This study
FR5851	FR5851	Illumina HiSeq	270	3863269		This study
	FR5851-P2M*	Illumina NextSeq	298	3854851		This study
FR5869	FR5869	Illumina HiSeq	270	3864372		This study
	FR5869-P2M*	Illumina NextSeq	288	3878775		This study

*-R1/-R2/-P2M/-P2M2: internal labels for biological replicates of sequencing using Illumina NextSeq technology.

Technical Appendix 1 Table 3. Outbreak genomes of *B. pertussis*

Isolate_Id	GenBank accession no.	Origin	Year of collection	Reference
CHLA-13	GCA_000479415.2	California	2010	(*24*)
CHLA-15	GCA_000479435.2	California	2010	(*24*)
CHLA-20	GCA_000479915.2	California	2012	(*24*)
CHLA-26	GCA_000479935.2	California	2010	(*24*)
H374	GCA_001605035.1	California	2010	(*19*)
H375	GCA_001605135.1	California	2010	(*19*)
H378	GCA_001605055.1	California	2010	(*19*)
H379	GCA_001605075.1	California	2010	(*19*)
H380	GCA_001605095.1	California	2010	(*19*)
H489	GCA_001605175.1	California	2010	(*19*)
H542	GCA_001605195.1	California	2010	(*19*)
H559	GCA_001605115.1	California	2010	(*19*)
H561	GCA_001605215.1	California	2010	(*19*)
H563	GCA_001605235.1	California	2010	(*19*)
H564	GCA_001605345.1	California	2010	(*19*)
H622	GCA_001605255.1	California	2010	(*19*)
H627	GCA_001605155.1	California	2010	(*19*)
H788	GCA_001605365.1	Vermont	2011	(*19*)
H918	GCA_000479475.2	Washington	2012	(*24*)
H921	GCA_000479495.2	Washington	2012	(*24*)
H939	GCA_000479575.2	Washington	2012	(*24*)
H973	GCA_000479595.2	Washington	2012	(*24*)
I002	GCA_000479395.2	Washington	2012	(*24*)
I036	GCA_000479515.2	Washington	2012	(*24*)
I176	GCA_000479535.2	Washington	2012	(*24*)
I468	GCA_001605385.1	Vermont	2012	(*19*)
I469	GCA_001605405.1	Vermont	2012	(*19*)
I472	GCA_001605425.1	Vermont	2012	(*19*)
I475	GCA_001605705.1	Vermont	2012	(*19*)
I476	GCA_001605445.1	Vermont	2012	(*19*)
I480	GCA_001605465.1	Vermont	2012	(*19*)
I483	GCA_001605485.1	Vermont	2012	(*19*)
I488	GCA_001601785.1	Vermont	2012	(*19*)
I496	GCA_001605505.1	Vermont	2012	(*19*)
I498	GCA_001605525.1	Vermont	2012	(*19*)
I517	GCA_001601775.1	Vermont	2012	(*19*)
I518	GCA_001605545.1	Vermont	2012	(*19*)
I521	GCA_001605565.1	Vermont	2012	(*19*)
I538	GCA_001605585.1	Vermont	2012	(*19*)
I539	GCA_001605605.1	Vermont	2012	(*19*)
I646	GCA_001605625.1	Vermont	2012	(*19*)
I656	GCA_001605645.1	Vermont	2012	(*19*)
I669	GCA_001605665.1	Vermont	2011	(*19*)
I707	GCA_001605685.1	Vermont	2012	(*19*)
STO1-CHLA-0006	GCA_000479555.2	California	2010	(*24*)
STO1-CHLA-0011	GCA_000479695.2	California	2010	(*24*)
STO1-CHOC-0008	GCA_000479795.2	California	2010	(*24*)
STO1-CHOC-0016	GCA_000479895.2	California	2010	(*24*)
STO1-CHOC-0017	GCA_000479755.2	California	2010	(*24*)
STO1-CHOC-0018	GCA_000479775.2	California	2010	(*24*)
STO1-CHOC-0019	GCA_000479835.2	California	2010	(*24*)
STO1-CHOC-0021	GCA_000479815.2	California	2010	(*24*)
STO1-CHOM-0012	GCA_000479855.2	Michigan	2010	(*24*)
STO1-CNMC-0004	GCA_000479875.2	Washington	2010	(*24*)
STO1-SEAT-0004	GCA_000479615.2	Washington	2011	(*24*)
STO1-SEAT-0006	GCA_000479715.2	Washington	2012	(*24*)
STO1-SEAT-0007	GCA_000479675.2	Washington	2012	(*24*)
2250905	GCA_000479635.2	California	2010	(*24*)
2356847	GCA_000479655.2	California	2010	(*24*)
2371640	GCA_000479735.2	California	2010	(*24*)
ERS176862	ERS176862	UK	2012	(*25*)
ERS176863	ERS176863	UK	2012	(*25*)
ERS176864	ERS176864	UK	2012	(*25*)
ERS176865	ERS176865	UK	2012	(*25*)
ERS176866	ERS176866	UK	2012	(*25*)
ERS176867	ERS176867	UK	2012	(*25*)
ERS176868	ERS176868	UK	2012	(*25*)

Isolate_Id	GenBank accession no.	Origin	Year of collection	Reference
ERS176869	ERS176869	UK	2012	(*25*)
ERS176870	ERS176870	UK	2012	(*25*)
ERS176871	ERS176871	UK	2012	(*25*)
ERS176872	ERS176872	UK	2012	(*25*)
ERS176873	ERS176873	UK	2012	(*25*)
ERS176874	ERS176874	UK	2012	(*25*)
ERS176875	ERS176875	UK	2012	(*25*)
ERS227757	ERS227757	UK	2012	(*25*)
ERS227758	ERS227758	UK	2012	(*25*)
ERS227759	ERS227759	UK	2012	(*25*)
ERS227760	ERS227760	UK	2012	(*25*)
ERS227761	ERS227761	UK	2012	(*25*)
ERS227762	ERS227762	UK	2012	(*25*)
ERS227763	ERS227763	UK	2012	(*25*)
ERS227764	ERS227764	UK	2012	(*25*)
ERS227765	ERS227765	UK	2012	(*25*)
ERS227766	ERS227766	UK	2012	(*25*)
ERS227767	ERS227767	UK	2012	(*25*)
ERS227768	ERS227768	UK	2012	(*25*)
ERS227769	ERS227769	UK	2012	(*25*)
ERS227770	ERS227770	UK	2012	(*25*)
ERS227771	ERS227771	UK	2012	(*25*)
ERS227772	ERS227772	UK	2012	(*25*)
ERS227773	ERS227773	UK	2012	(*25*)
ERS227774	ERS227774	UK	2012	(*25*)
ERS227775	ERS227775	UK	2012	(*25*)
ERS227776	ERS227776	UK	2012	(*25*)
ERS227777	ERS227777	UK	2012	(*25*)
ERS227778	ERS227778	UK	2012	(*25*)
ERS227785	ERS227785	UK	2012	(*25*)
ERS227786	ERS227786	UK	2012	(*25*)
ERS227787	ERS227787	UK	2012	(*25*)
ERS227788	ERS227788	UK	2012	(*25*)
ERS227789	ERS227789	UK	2012	(*25*)
ERS227790	ERS227790	UK	2012	(*25*)
ERS227791	ERS227791	UK	2012	(*25*)
ERS227792	ERS227792	UK	2012	(*25*)
ERS227793	ERS227793	UK	2012	(*25*)
ERS227794	ERS227794	UK	2012	(*25*)
ERS227795	ERS227795	UK	2012	(*25*)
ERS227796	ERS227796	UK	2012	(*25*)
ERS227797	ERS227797	UK	2012	(*25*)
ERS227798	ERS227798	UK	2012	(*25*)
ERS227799	ERS227799	UK	2012	(*25*)
ERS227800	ERS227800	UK	2012	(25)
ERS227801	ERS227801	UK	2012	(*25*)

Technical Appendix 1 Table 4. NCBI genomes of *B. pertussis* isolates used to define the core genome (from https://www.ncbi.nlm.nih.gov/genome/genomes/1008)

Name	Accession no.
18323	GCA_000306945.1
2250905	GCA_000479635.2
2356847	GCA_000479655.2
2371640	GCA_000479735.2
18323–2	GCA_001525555.1
ATCC BAA-1335D-5	GCA_001558395.1
B1838	GCA_001307585.1
B1865	GCA_001307605.1
B1917	GCA_000193595.3
B200	GCA_000662235.1
B3405	GCA_001307625.1
B3582	GCA_001307645.1
B3585	GCA_001307665.1
B3621	GCA_001307565.1
B3629	GCA_001307525.1
B3640	GCA_001307685.1
B3658	GCA_001307705.1
B3913	GCA_001307725.1
B3921	GCA_001307745.1
Bp137	GCA_000812165.1
BpC90	GCA_001187405.1
C393	GCA_001605275.1
CHLA-11	GCA_000504325.1
CHLA-13	GCA_000479415.2
CHLA-15	GCA_000479435.2
CHLA-20	GCA_000479915.2
CHLA-26	GCA_000479935.2
Cs	GCA_000212975.1
E476	GCA_001605295.1
H374	GCA_001605035.1
H375	GCA_001605135.1
H378	GCA_001605055.1
H379	GCA_001605075.1
H380	GCA_001605095.1
H489	GCA_001605175.1
H542	GCA_001605195.1
H559	GCA_001605115.1
H561	GCA_001605215.1
H563	GCA_001605235.1
H564	GCA_001605345.1
H622	GCA_001605255.1
H627	GCA_001605155.1
H788	GCA_001605365.1
H897	GCA_000479455.2
H918	GCA_000479475.2
H921	GCA_000479495.2
H934	GCA_000662295.1
H939	GCA_000479575.2
H973	GCA_000479595.2
I002	GCA_000479395.2
I036	GCA_000479515.2
I176	GCA_000479535.2
I468	GCA_001605385.1
I469	GCA_001605405.1
I472	GCA_001605425.1
I475	GCA_001605705.1
I476	GCA_001605445.1
I480	GCA_001605465.1
I483	GCA_001605485.1
I488	GCA_001601785.1
I496	GCA_001605505.1
I498	GCA_001605525.1
I517	GCA_001601775.1
I518	GCA_001605545.1

Name	Accession no.
I521	GCA_001605565.1
I538	GCA_001605585.1
I539	GCA_001605605.1
I646	GCA_001605625.1
I656	GCA_001605645.1
I669	GCA_001605665.1
I707	GCA_001605685.1
STO1-CHLA-0006	GCA_000479555.2
STO1-CHLA-0011	GCA_000479695.2
STO1-CHOC-0008	GCA_000479795.2
STO1-CHOC-0016	GCA_000479895.2
STO1-CHOC-0017	GCA_000479755.2
STO1-CHOC-0018	GCA_000479775.2
STO1-CHOC-0019	GCA_000479835.2
STO1-CHOC-0021	GCA_000479815.2
STO1-CHOM-0012	GCA_000479855.2
STO1-CNMC-0004	GCA_000479875.2
STO1-SEAT-0004	GCA_000479615.2
STO1-SEAT-0006	GCA_000479715.2
STO1-SEAT-0007	GCA_000479675.2
